# A Global Expression Switch Marks Pachytene Initiation during Mouse Male Meiosis

**DOI:** 10.3390/genes1030469

**Published:** 2010-12-13

**Authors:** Mohammad Fallahi, Irina V. Getun, Zhen K. Wu, Philippe R.J. Bois

**Affiliations:** Genome Plasticity Laboratory, Department of Cancer Biology, The Scripps Research Institute, Scripps Florida, Jupiter, FL 33458, USA; E-Mail: mfallahi@scripps.edu (M.F.); igetun@scripps.edu (I.V.G.); zhenkwu@scripps.edu (Z.K.W.)

**Keywords:** meiosis, commitment, transcription, dynamic, expression array, mouse, MSCI

## Abstract

Male spermatogenesis is an essential and complex process necessary to gain totipotency and allow a whole new organism to develop upon fertilization. While single-gene based studies have provided insights into the mechanisms underlying spermatogenesis, detailed global profiling of all the key meiotic stages is required to fully define these processes. Here, by isolating highly enriched mouse meiotic cell populations, we have generated a comprehensive gene expression atlas of mammalian meiosis. Our data define unique signatures for the specific stages of meiosis, including global chromosome X inactivation and reactivation. The data also reveal profound switches in global gene expression at the initiation of pachynema that are reminiscent of the commitment to meiosis observed in budding yeast. Overall, this meiotic atlas provides an exhaustive blueprint and resource for mammalian gametogenesis and meiosis.

## 1. Introduction

Spermatogenesis is a complex developmental process that resets the entire genome. Spermatogenesis initiates with the establishment of germ cells in the genital ridges and culminates with the production of fully differentiated sperm [1,2]. While single-gene based studies have provided fundamental insights into some of the mechanisms that are operational during spermatogenesis, these processes are generally poorly defined. A major hurdle for the meiosis field has been the difficulty in isolating cells from the different stages of meiosis from the mammalian testis [3,4,5], in particular stages from pre-leptonema to middle pachynema. This has precluded a more global and detailed analysis of meiosis that would allow for the discovery of new developmental pathways, the identification of new stage specific markers, an understanding of changes that occur to the transcriptome, and insights into the regulatory mechanisms that control meiosis. 

Spermatogenesis requires complex interactions between the various compartments of the testis that allow germ cells to engage in meiosis and become spermatozoa. Meiotic cell division is unique to germ cells, is absolutely required for the production of haploid gametes, and is essential to maintain genomic integrity. Unlike somatic cell differentiation, where pluripotent cells are progressively locked into more restricted and highly specialized fates, germ cells go through a series of epigenetic events unique to this cell type that preserve totipotency and allow a whole new organism to develop upon fertilization and fusion of oocyte and spermatozoa to form a diploid zygote. The halving of chromosome number occurs in the prolonged prophase of meiosis I, which can be divided into distinct substages defined by distinct chromosomal events [6]. At the initiation of meiosis, sister chromatids replicate and enter prophase I at leptonema. The axial element, a proteinaceous backbone, immediately forms along each chromatin pair. It joins the two sister chromatids in a linear array that organizes chromatin into large 150–250 kb loops that extend out from the axial element. These homolog interactions condense the paired chromosomes and totally reorganize the architecture of the nucleus.

Double strand breaks (DSB) directed by the Spo11 meiotic-specific endonuclease initiate at leptonema and these are then bound by proteins that recognize and organize these single-stranded DNA ends at the zygonema stage, forming foci at recombination nodules. During this stage a chromosomal “bouquet” is observed, where telomeres, attached to the nuclear envelope, congregate opposite to the centrosome [7]. This contrasts with mitosis in somatic cells where centromeres generally point towards the centrosome. As homologous chromosomes align they become closely joined at discrete regions and then the final component, the synaptonemal complex (SC), zips the homologs together, leading to full synapsis during pachynema. At this stage, chromosomes are condensed and individual homologs are indistinguishable from their homologous partner. The exception are the X and Y chromosomes, which only synapse along their pseudo-autosomal region, and undergo large-scale chromatin remodeling and quasi-complete silencing in a peripheral nuclear subdomain called the sex- or XY-body in a processed called meiotic sex chromosome inactivation (MSCI) [8,9]. MSCI is thought to persist throughout the rest of prophase I. During diplonema, the SC disintegrates and homologous chromosomes begin to move apart, revealing individual chiasmata produced by recombination that function to hold the homologs together until the chromosomes are appropriately aligned. Chiasmata are then severed in anaphase I to allow the first meiotic division (MI) to proceed, which segregates the homologs to opposite poles of the cell to produce two daughter cells that then enter meiosis II.

To obtain a precise atlas of this complex process, we refined a FACS based methodology [10,11] that allows one to isolate all of the principal stages of meiosis, from pre-meiotic spermatogonia to haploid round spermatids, with purities greater than 95–98%. Expression array analysis and validation of these arrays has provided a detailed atlas of the changes in gene expression that are manifest during this unique development process. Our data are, to the best of our knowledge, the most refined and detailed to date to provide a global expression blueprint of male mouse meiosis. These data also validate the robust and facile FACS-based methodology as a technique to reliably and quickly isolate highly pure meiotic fractions from adult mouse testis. Most importantly, these studies have defined the dynamics of silencing and partial reactivation of chromosome X (chr X) during meiosis I progression and have also revealed a profound switch in the transcriptome at the early pachynema stage that is reminiscent of the point of commitment to meiosis observed in yeast [12]. 

## 2. Results and Discussion

### 2.1. Meiotic Cell Purification

**Figure 1:**
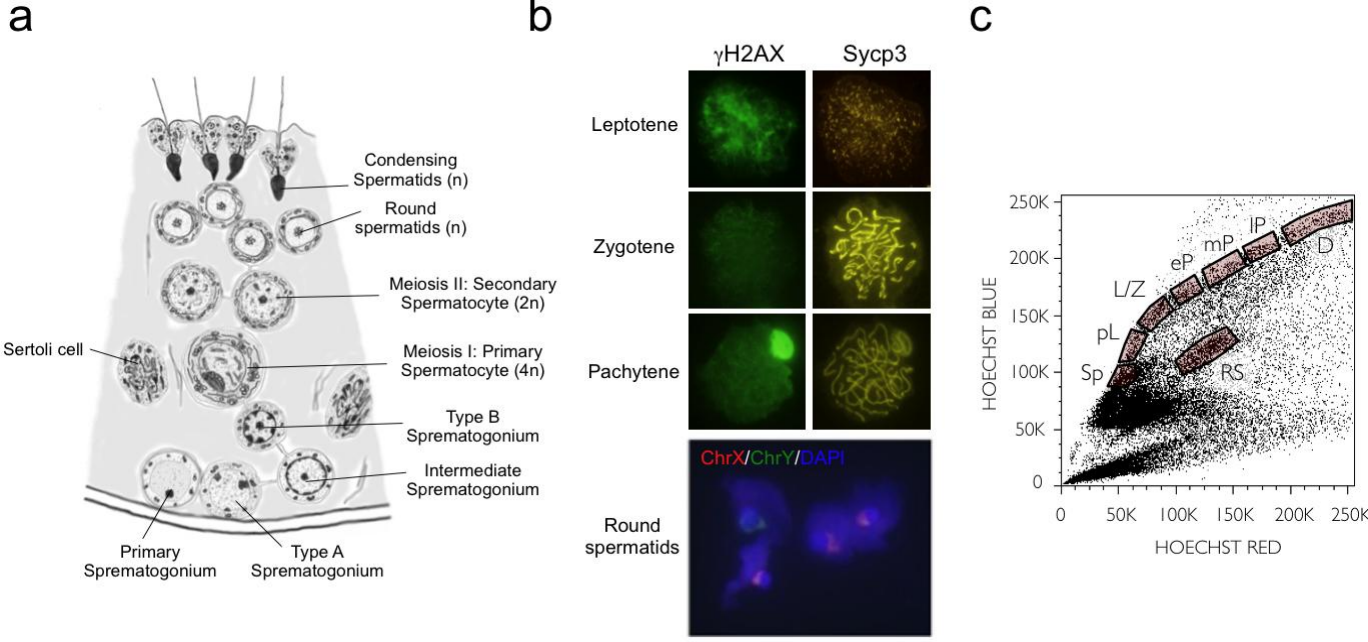
Architecture of the mammalian testis and purification of meiotic fractions. (**a**) Schematic drawing of a section of a seminiferous tubule with mitotic spermatogonia, spermatocytes, and spermatids surrounded by two Sertoli nurse cells; (**b**) Representative pictograms of FACS-purified meiosis prophase I meiotic stages labeled for phosphorylated histone H2AX and synaptonemal complex protein 3 (immunofluorescence analyses), as well as the split sexual chromosome in round spermatids (fluorescent *in situ* hybridization); (**c**) A representative Hoechst FACS profile is shown, with the various meiotic cell populations used in this study indicated: spermatogonia (Sp), pre-leptotene (pL), leptotene-zygotene (L/Z), early-pachytene (eP), middle-pachytene (mP), late-pachytene (lP), diplotene (D), and round spermatids (RS).

Germ-cell development is unique in that it gives rise to an entire organism. Developmentally, cells undergoing meiosis originate from a population of pluripotent cells in the epiblast, which give rise to primordial germ cells (PGCs) [1]. In mice, a cluster of cells emerges inside the extra-embryonic mesoderm (E7.25) and their genome then undergoes epigenetic modifications that suppress somatic programming. PGCs then migrate (E10.5) and settle in the genital ridge (E11.5) where erasure and establishment of parental imprints proceeds (for review see Sasaki *et al.* [2]). In males at puberty, G1 arrested PGCs are re-activated, leading to spermatogonial self-renewal, meiosis, and spermiogenesis. This is a highly ordered process where mitotic proliferation of germ stem cells is sustained through type A spermatogonia, intermediate spermatogonia, and then type B spermatogonia. The latter then engage in meiosis to form pre-leptonema primary spermatocytes that undergo a final S-phase before entering meiotic prophase. These cells are intercalated between adjacent Sertoli cells on the adluminal side of the intercellular Sertoli junction and it is here that equational and reductional meiotic divisions take place to form haploid spermatids. Finally, differentiation is completed by spermiogenesis, which is a complex morphological transformation that culminates with the release of late spermatids into the lumen of the seminiferous tubule (Figure 1).

To obtain gene expression profiles for each of the major stages of mammalian male meiosis, we refined a FACS methodology using Hoechst 33342 stain that allows one to purify various meiotic fractions from adult male dissociated testicular cells [10,11,13,14] (see Methods section), and obtained very robust and reliable profiles for each of the major stages of meiosis (Figure 1c). This method combines the red and blue fluorescence of the Hoechst 33342 vital dye to discriminate cell DNA content (ploidy) and the efficiency of DNA staining, which varies according to the extent of chromatin condensation in various staged meiotic cells. As germ cells progress through meiosis, their DNA content is diploid (spermatogonia and secondary spermatocytes), tetraploid (leptonema, zygonema, pachynema, and diplonema) or haploid (round spermatids). We confirmed the correct stage and level of purity of each of these sorted fractions by immunohistochemistry (Figure 1b) [14]. Meiotic-specific markers of the synaptonemal complex protein SCP3 and histone γH2AX were used to discriminate the purity of the pre-leptotene to diplotene stage meiotic cells, and round spermatids were verified using X and Y specific chromosome painting. These analyses established a high degree of enrichment, above 95–98%, for each FACS sorted fraction, excluding the leptotene/zygotene fraction where approximately 50% of each stage were detected. Importantly, minor contaminating cells in each fraction were strictly meiotic in nature from adjacent fractions, with no evidence of somatic cell contamination. 

Independent biological samples, comprised of pools of at least three independent FACS sorts for each meiotic population, were then analyzed for their global transcriptomes using Affymetrix MOE430 version 2 microarray. The gene expression patterns from these 20 microarrays provided a global and detailed atlas of the dynamics of gene expression during meiotic progression.

### 2.2. Expression Analysis Overview

**Figure 2:**
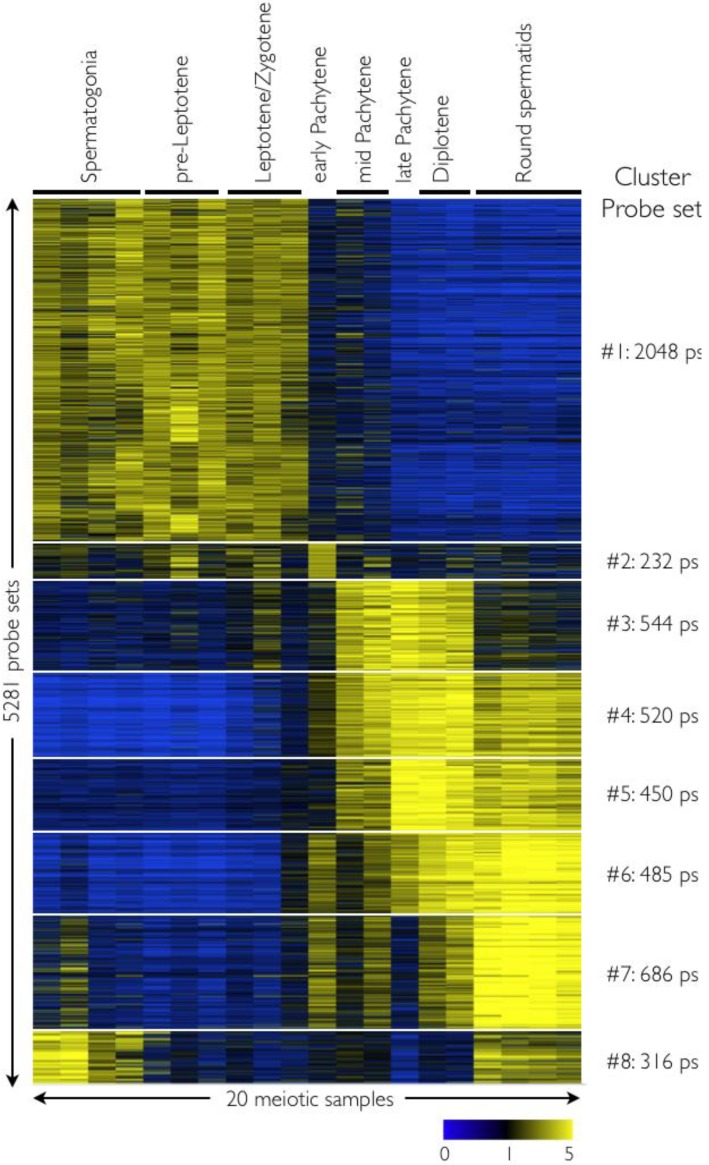
Dynamics of meiotic gene expression. The heatmap shows probe sets having meiotic fraction-specific genes, which are indicated. Each horizontal line corresponds to a probe set with yellow and blue indicating normalized high and low expression, respectively. Genes are divided into 8 K-means clusters. Complete gene lists and GO terms tables are available in the Supporting Information (Supplementary Dataset S1 and Dataset S2).

A combination of comparative and analytical approaches was used to identify and derive informative gene expression signatures for all stages of mouse spermatogenesis. One objective was to obtain an overview of the dynamics of global gene expression between the closely related meiotic populations. Initially, 20 microarray GeneChips were subjected to MAS5 detection call algorithm (Affymetrix Expression Console v1.0) to identify probe sets that were present (*p* < 0.05) in each population. For populations with more than two chip replicates, one wild card detection call was used. To define the most stage-specific genes, probe sets designated as “Present” were ranked based on their average GC-RMA signals normalized to the median across all 20 arrays, and the 1,000 top-ranked probe sets for each population were identified and pooled together using GeneSpring GX 7.3.1. This revealed 5,281 (5.3 K) probe sets, which we subjected to K-means clustering. This yielded 8 sets that had clear meiotic fraction-specific signatures (Figure 2). Finally, enriched biological processes within each cluster were identified using GO ontology terms. 

These heatmaps revealed an expected relatedness between several meiotic fractions, where populations prior to mid-pachynema are closely related. For example, the largest cluster #1, having 2,048 shared probe sets, is observed from spermatogonia to leptonema/zygonema. Indeed, as one might predict, gene set enrichment analysis (GSEA) revealed that many of these genes are involved in spindle regulation, mitosis, helicase activity, sexual reproduction and gamete generation (Supplementary Figure S1) [15,16]. Importantly, however, these datasets also revealed a striking switch in the transcriptome between the leptonema/zygonema and mid-pachynema stages. Indeed, the signature of the early pachynema fraction indicates global reprogramming of gene expression at this interval of sperm development (Figure 2, and see below). The complete probe set list of each cluster is provided in Supplementary Dataset S1 and detailed gene ontology enrichment analyses for the set of genes found in each cluster are presented in Supplementary Dataset S2.

To validate our dataset, we also performed clustering analysis by comparing our datasets with those from two other previously performed studies [4,5]. These datasets were both collected using the identical Affymetrix platform, allowing efficient comparison. The first dataset collected by the Primig laboratory (European Bioinformatics Institute ArrayExpress public data repository, E-TABM-130) consists of sertoli cells (>95% purity), spermatogonia (>85%), pachytene spermatocytes (>82.5%), round spermatids (90–95%), tubules, and whole testis [5]. The second dataset was collected by the Lee laboratory (Gene Expression Omnibus, GSE4193) from four meiotic subpopulations including spermatogonia type A and B (>85%), pachytene spermatocytes (>95%), and round spermatids (>95%) [4]. Clustering analysis produced the expected distribution with our purified meiotic populations found between the two other datasets (Supplementary Figure S2 and Figure S3). Moreover, we found strong correlation between closely related populations (Supplementary Table S1).

### 2.3. Meiotic Sex Chromosome Inactivation

**Figure 3:**
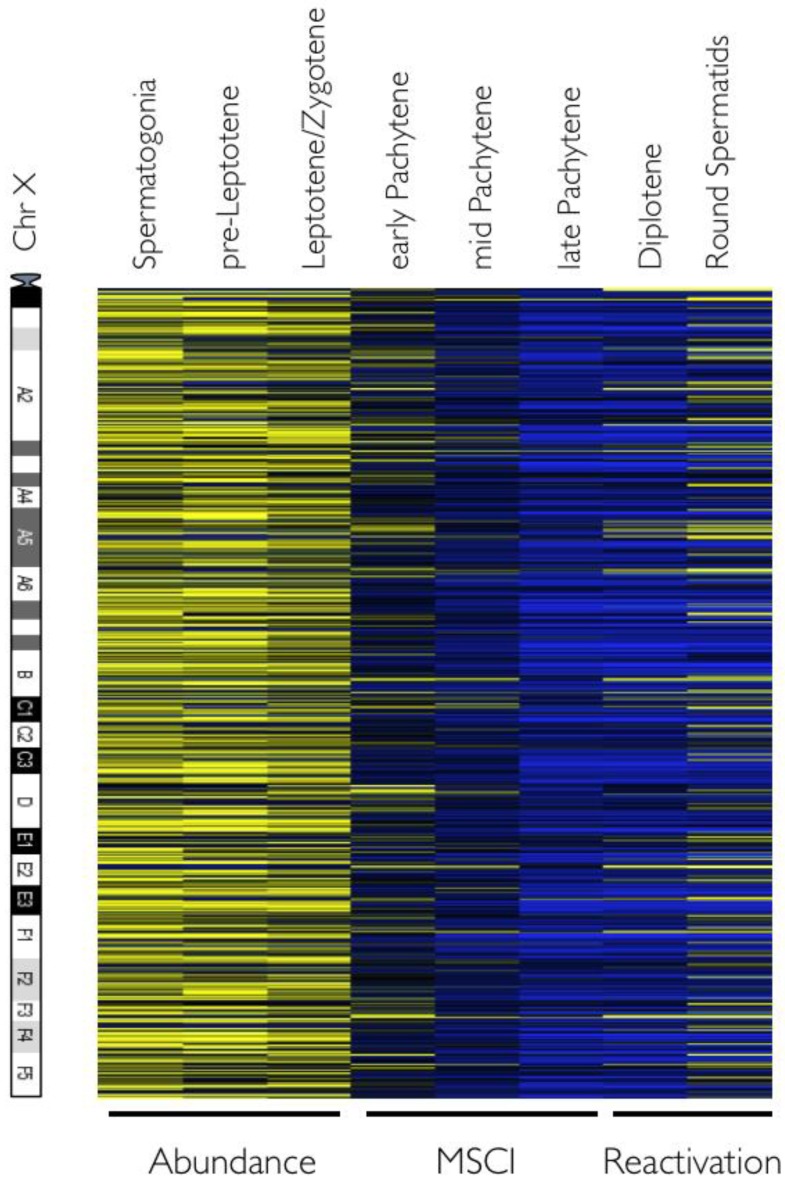
Meiotic sex chromosome inactivation and reactivation. The average expression of 874 X-linked present probe sets is shown. Complete inactivation is clearly visible at the late pachynema stage. The three phases of chr X meiotic developmental stage are indicated below the heatmap. Probe sets were serially ordered relative to their chromosome location from centromere to telomere but are not to scale. A schematic of chr X is shown on the left for orientation purposes.

We analyzed the dynamics of chr X inactivation and reactivation, a feature unique to male meiosis, where both unsynapsed sexual chromosomes are subjected to global epigenetic regulation that directs silencing across chr X [17]. To this end, we generated a heatmap of 874 X-linked probe sets (513 unique and 48 multicopy genes) and these were ordered relative to their chromosome location from centromere to telomere (Figure 3). As previously noted, chr X gene expression is elevated in the early steps of spermatogenesis, leading to a very significant overall bias toward X-link probe sets (*p* = 3.09 × 10^−50^) [18,19]. As expected, as soon as early pachynema is initiated or at the zygonema-to-pachynema transition, chr X is rapidly silenced and set apart in the sex-body (Figure 1b) [8]. There was an average 12-fold reduction in probe set signal between spermatogonia and late pachynema, where chr X transcription is at its lowest. MSCI has been thought to persist throughout the rest of prophase I (pachynema and diplonema) [4,19], yet our refined dataset established that this is not the case. Indeed, partial chr X reactivation is seen for some genes as early as diplonema. This strongly suggests that a complete MSCI is a rather transient state, echoing observations demonstrating that some regions of chr X may escape inactivation [20]. This reactivation, while far from complete, still represents a highly significant proportion or 37.7% (330 probe sets) of the total probe sets initially selected. Most interestingly, when analyzing outliers, most were due to curation errors where they were assigned to the wrong chromosome all together, or represented probe sets present in multiple copies across the mouse genome, for example those encoding highly expressed ribosomal proteins (Rps7, Rps20, Rps28, Rpl3) or Odc1 (Ornithine decarboxylase 1) and its many pseudogenes. Overall, a total of 15 probe sets had to be excluded. However, some were *bona fide* X-linked genes such as *Akap4* (A-kinase anchoring protein 4), a gene encoding a scaffold for complexes involved in regulating flagella function [21,22], and Prdx4 (Peroxiredoxin 4), which is also essential for spermatogenesis [23]. These may either escape MSCI or their expression may reflect differences in mRNA turnover. Indeed, there was a good correlation between pre-pachynema expression level and level present at late pachynema (Pearson correlation *r*_spermatogonia *vs.* late pachytene_ = 0.69). In fact, the most highly expressed chr X transcripts at the spermatogonia stage (10-fold higher than average) were still subject to >10-fold repression at late pachynema. Thereafter, these genes were robustly expressed, with an average 5.25-fold induction between late pachynema and round spermatids, restoring levels to half of their initial levels in spermatogonia. A complete X-linked probe set list is provided in Supplementary Dataset S3.

### 2.4. Commitment to Meiosis

Our finding of an abrupt switch in global gene expression at the beginning of pachynema was intriguing. Indeed, unlike the repetitive mitotic cell cycle, meiosis is a terminal, linear process that culminates with the production of fully formed spermatids. At one stage, a choice has to be made between meiotic or mitotic cycle. This switch of expression may correspond to a point of no return and represent commitment towards gamete formation. Intriguingly, the timing of this switch is very similar to what has been described in budding yeast where commitment to meiosis represents a point where they cannot return to the mitotic cycle even when receiving cues (*i.e*., nutrients) that drive proliferation (return to growth) [12]. Yeast become fully committed to meiosis at the end of prophase I, long past DNA replication (pre-leptonema), DSB initiation, and establishment of the SC, yet before the first meiotic division. This scenario appears conserved, given the timing of the switch in global gene expression during meiosis. 

**Figure 4:**
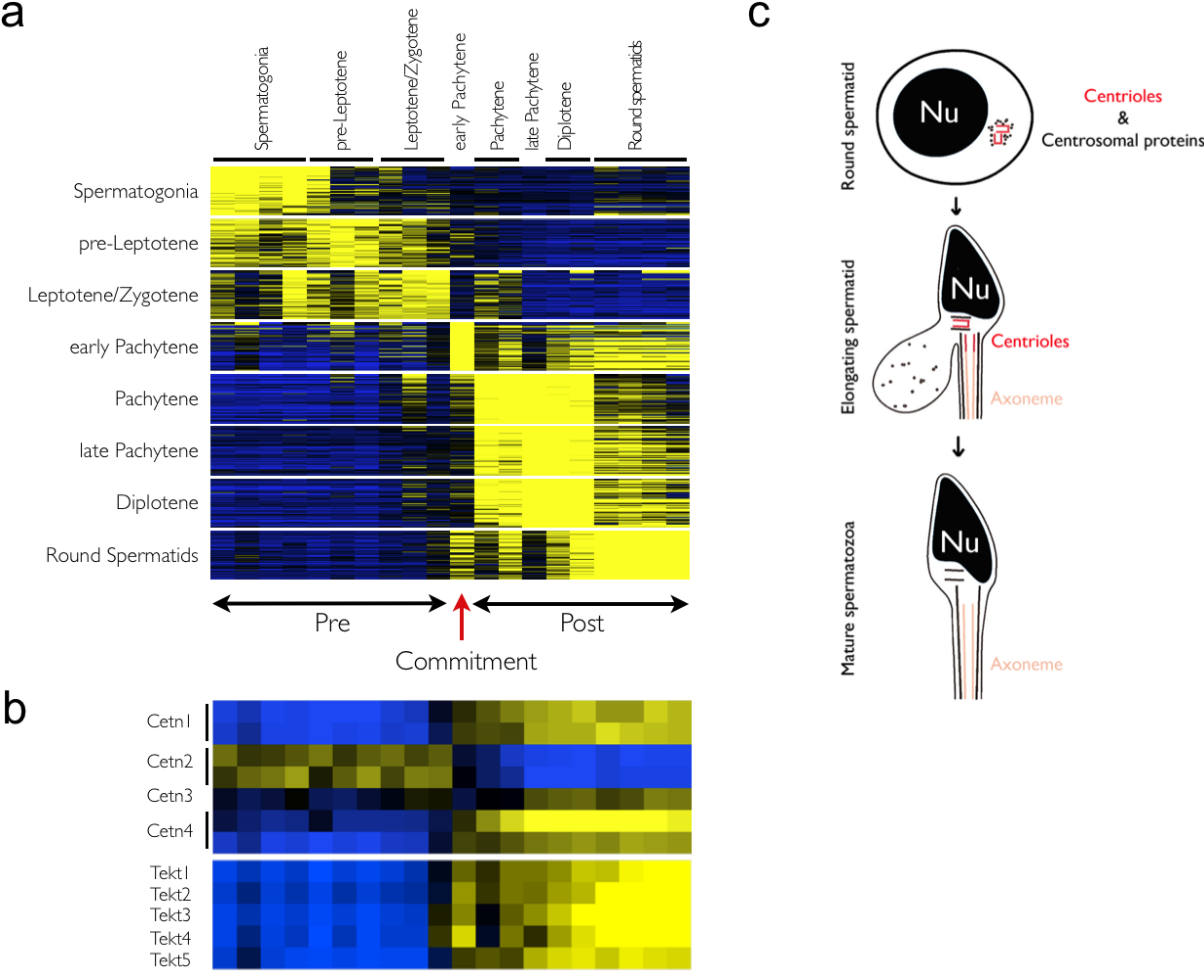
Meiotic fraction-specific genes and commitment. (**a**) The top 50 probe sets having the most restricted expression patterns are shown for each of the eight meiotic fractions studied. A high degree of correlative gene expression is observed in cell populations before and after the early-pachytene switch; (**b**) Heatmaps of centrin and tektin probe sets are displayed. An expression switch is observed suggesting a totally new regulation of these fundamental regulators of centrioles; (**c**) Centrosome reduction during spermiogenesis. The male germ cells possess intact centrosomes containing centrioles and centrosomal proteins until the round spermatid stage. The microtubules of the distal centriole extend as axoneme of the spermatid tail. The centrioles are degenerated to various extents in spermatozoa of different species. Rodent spermatozoa lose both centrioles completely (shown here). The schematics are not drawn to scale (adapted from Manandhar *et al.* [24]). Nu: nucleus.

To confirm and further define this global reprogramming of the transcriptome, additional *in silico* analyses were performed by selecting the top 50 most specific probe sets from each meiotic population analyzed (Figure 4a). These analyses confirmed that many genes are expressed in adjacent fractions prior to and following early pachynema, with a clear transition at this central point. In yeast, the duplication of spindle-pole bodies (SPBs) triggers commitment [12]. SPBs are similar to centrioles in animal cells, which are essential for the formation of microtubule-derived structures including centrosomes and flagella [25]. Unfortunately, little is known about the regulation of centriole duplication in male meiosis. During meiosis, there is an uncoupling of chromosome and centrosome cycles, where centrioles duplicate from meiosis I to meiosis II without DNA replication [24], and in sperm differentiation one of the centrioles matures into the flagella, a hallmark of developed spermatids (Figure 4c). Centrin is a key regulator of centriole duplication [26]; with humans and mice having four centrin genes that are expressed in a cell-type specific manner, where *Cetn2* and *Cetn3* are expressed in all somatic cells, *Cetn1* in male germ cells and certain neurons, and both *Cetn1* and *Cetn4* in ciliated cells. Expression analysis of the four mammalian centrins revealed an interesting switch precisely at the early pachynema transition, where the uniquely expressed *Cetn2* was repressed and robust expression of the three other centrins was initiated (Figure 4b). Similarly, centrosome-specific tektins [27], important components of flagella, show the same pattern of regulation, again suggesting that a unique transition happens at this specific stage of meiosis. It thus appears that, similar to yeast, reprogramming of centriole, and more generally, centrosome regulation, is directed by a switch in the expression of centrins (a complete list of the probe sets used in Figure 4 are provided in Supplementary Dataset S4).

### 2.5. Discussion

The expression atlas of mouse spermatogenesis provided herein establishes a foundation for the global analysis of mammalian meiosis, akin to that performed for other organs [28,29]. Such analyses offer numerous advantages over *in situ* hybridization only strategies that are generally qualitative. Importantly, our analyses of FACS-purified meiotic stage cells revealed tight correlations between replicates of these cell populations, resulting in homogeneous profiles. Finally, these analyses should allow one to rigorously interrogate conserved aspects of gametogenesis, by comparison to other meiotic models (e.g., *Drosophila*, *C. elegans*, and human).

This expression atlas extends prior microarray analyses of spermatogenesis, which analyzed testis development [3,4,5], and their combination now provides a rich resource to mine and build upon. Some of these studies took advantage of the semi-synchronous nature of the “first wave” of meiosis in male mice from seminiferous tubules that principally contain gonocytes, spermatogonia, and Sertoli cells at Day one, which are rapidly outnumbered by differentiating germ cells as the animal ages [30]. However, our analysis by FACS of pre-puberal mice, as well as molecular analysis, indicate that there is no such distinct wave and that such data should be analyzed cautiously [31]. More refined, yet partial transcriptional analyses were also reported using purified spermatocytes (e.g., spermatogonia, pachynema stage, and round spermatids) using gravity sedimentation or elutriation [4,32]. However, the study presented here defines a comprehensive microarray analysis of mammalian meiosis progression using FACS methodology as an efficient and simple purification strategy. Interestingly though, previous datasets showed excellent clustering with our entire panel (Supplementary Figure 2 and Figure 3, Supplementary Table 1) [4,5], even those performed with previous versions of Affymetrix microarrays (data not shown) [3].

Our analysis of chr X inactivation [17], a truly unique feature of male meiosis, validates the quality of this expression atlas. Indeed, visualizing the extinction of the entire chr X can only be observed if contaminating cells are not present (Figure 3). As previously shown, early stage meiotic cells do express high levels of X-linked genes, which leads to their over representation in spermatogonia [18]. These analyses revealed that chr X inactivation is indeed global, and they suggest that chr X transcripts remaining in late pachynema likely have long half-lives rather than being escapees from MSCI. Surprisingly, our analyses failed to confirm previous findings suggesting a bias for elevated expression of X-linked multicopy genes following meiosis [33]. Indeed, when the signals of multicopy chr X genes were normalized to their published copy number [33], they were, on average, expressed almost two-fold lower than single copy chr X genes (Supplementary Dataset S4).

Previous microarray analyses in mouse, rat, and human suggested two core transcriptional programs in pre- *versus* post-meiotic cells [4,5]. However, due to the rather low resolution of these studies, it was not clear if this was a gradual or abrupt switch in gene expression. Our data suggest that an abrupt and global meiotic expression transition occurs at early pachynema. The mechanisms controlling this striking transition in germ cell expression profiles remain to be defined. Here, we speculate that this corresponds to a developmental stage where the commitment to spermiogenesis is made. This cell fate commitment is a complex process and it is likely that different aspects of meiosis are involved at different stages. In support of this notion, specific developmental arrest points at distinct intervals of spermatogenesis have been established in knockout and transgenic mice, from the proliferation of PGCs to spermatid development [34]. Interestingly, mice deficient in DNA damage/repair (e.g., Spo11, Dmc1, Msh4, and Mlh1) or in the establishment of SC (e.g., Sycp3) fail to progress beyond zygonema and present a pseudo-pachynema phenotype, which precisely corresponds to the time when the expression profile transition is observed. Finally, the observed switch in the expression of centrins and tektins that is manifest at this stage (Figure 4b) suggests that structural components may impose this point of non-return, as seen in yeast [12].

## 3. Experimental Section

### 3.1. Mice

Male C57Bl/6JxDBA/2J F1 mice at 10–14 weeks of age were analyzed. The Scripps Florida Institutional Animal Care and Use Committee approved all procedures. 

### 3.2. Sample Collection and RNA Extraction

Meiotic cell fractions were purified by refining a previously described FACS-based methodology [10,11]. Proper cell dissociation is the most critical step and we adapted a previously described protocol [13,14]. Briefly, decapsulated testis were incubated for 15 min in 3 mL of Gey’s Balanced Salt Solution (GBSS) containing 10 µg of DNAse I (added at each step) and 1.5 mg of Collagenase Type I, and agitated at the constant 120 rpm, 33 °C. Dispersed seminiferous tubes were left to sediment for 1–3 min at room temperature, and the supernatant was discarded. This step was repeated twice. Seminiferous cells were then incubated under identical agitation condition in 3 mL of GBSS containing Collagenase, and 2.5 mg of Trypsin for 15 min at 33 °C. Repeated pipetting gently separated aggregates. Dispersed seminiferous cells were further incubated at the same shaking and temperature conditions for 10 min after adding DNAse I, Trypsin, and 0.4 mg of Hoechst 33342 as a pre-stain step. Following complete dissociation, 500 µL of FCS was added to inactivate trypsin. Staining was performed by adding DNAse I and 0.5 mg of Hoechst 33342 for 8 min and then propidium iodine was added to the stained cells, which were then filtered through 40 µm strainers. 

Sorting was performed using a Becton-Dickinson Aria IIu cell sorter. We used conditions similar to those previously described [10,11], but adapted these conditions for violet light since the Aria IIu lacks a UV laser. Cells were kept on ice and protected from light until sorting. Usually, three to four testes were processed per sort. RNA was prepared with RNAqueous Micro kits (Ambion AM1931). Each independent RNA pool included cells from multiple sorts of meiotic cells from mice. 

### 3.3. Immunofluorescence Analyses

Immunofluorescence analyses were performed as previously described [35]. Images were captured using an Olympus BX61 microscope with BX-UCB DP manager/controller. Primary antibodies were anti-SCP3 (Abcam, ab15092) and anti-phospho-histone H2AX-Ser139, clone JBW301 (Millipore, 05-636). Secondary antibodies used were goat anti-rabbit Cy3 (Jackson ImmunoResearch Laboratories, 111-165-144) and goat anti-mouse FITC (Abcam, ab6785). Mouse chromosome X (red, lot #080160) and Y (green, lot #080160) specific FISH probes were purchased from Applied Spectral Imaging and used as per the supplier’s instructions.

### 3.4. RNA Labeling and ARRAY Hybridization

The Genomics Core Facility of Scripps Florida ran the arrays using 0.2–1.0 μg of total RNA. Biotin-labeled cRNA probes were prepared as recommended by the manufacturer (Affymetrix), with oligo-dT primers, SuperScript, and BioArray High Yield RNA Transcript kits (Affymetrix). Fragmentation/hybridization to MOE430v2.0 arrays (Affymetrix) were performed according to Affymetrix protocols. Imaging was performed on a Hewlett Packard GeneArray Scanner. MIAME-compliant raw data has been deposited into the NCBI Gene Expression Omnibus database (see below). Microarray QC metrics such as scaling factor, percent of Present genes, and housekeeping/spike-in control genes 3'/5' signal ratios all passed the core facility standards. The microarray data are available in the Gene Expression Omnibus (GEO) database (http://www.ncbi.nlm.nih.gov/gds) under the accession number GSE21447. Additionally, data can be quickly accessed from the laboratory web site (http://www.scripps.edu/bois).

### 3.5. Data Analysis

A list of the most stage-specific genes out of all genes defined as being ‘Present’ in at least in one population was generated (see Results). K-means clustering (100 iterations, Pearson correlation, average linkage) in GeneSpring GX 7.3.1 (Agilent) was used to partition the above list into eight clusters. Enriched gene ontologies (Benjamini *p* < 0.05) were identified using GeneSpring GX. Gene Set Enrichment Analysis (GSEA) [15,16] tool determines whether a defined gene set shows statistically significant and concordant differences between two microarray datasets. The statistical result is summarized using FDR q-value. Briefly, GC-RMA signals were imported into GSEA tool. The number of permutations were set to 1,000, dataset was collapsed to gene symbols (max_probe), gene sets database was set to c5.all.v2.5.symbols.gmt [all gene ontologies], permutation type was set to gene_set, chip platform was set to Affymetrix MOE430v2.0, metric for ranking genes was set to Signal2Noise and the rest of the parameters were set to the default settings. For chr X analyses, *per* gene normalized GC-RMA signals were calculated and pooled for each population using GeneSpring GX. Affymetrix MOE430v2.0 annotation file was used to identify X-linked probe sets. Only probe sets defined as ‘Present’ in at least one population were visualized. Probe sets were ordered based on their chromosome location from centromere to telomere.

To evaluate the similarity of our data with previously published studies, two datasets were downloaded from public repositories (see Expression Analysis Overview). A total of 40 Affymetrix CEL files were normalized using GeneSpring GX GC-RMA algorithm. Probe sets with signals higher than the median in half of the samples (20) were used in the clustering analysis. Replicate samples were grouped for each cell type (total of 18 cell types). The GeneSpring GX hierarchical clustering algorithm was applied to the pooled samples. Distance metric was set to Pearson Centered and linkage rule was set to Average.

## 4. Conclusions

In summary, we provide a detailed gene expression atlas of mammalian male meiosis, which was generated using FACS based cell purification methods followed by hybridization to microarrays. This resource provides a valuable tool to further understand the complexity of the unique and remarkable processes that are operational during meiosis. Our findings also validate an efficient method to purify key stages of meiosis at high purity. 

## Supplementary Files

Supplementary File 1 Supplementary Dataset S1 (XLS, 2393 KB)

Supplementary File 2 Supplementary Dataset S2 (XLS, 1109 KB)

Supplementary File 3 Supplementary Dataset S3 (XLS, 295 KB)

Supplementary File 4 Supplementary Dataset S4 (XLS, 214 KB)

Supplementary File 5 Supplementary Figure S1 (TIF, 7141 KB)

Supplementary File 6 Supplementary Figure S2 (TIF, 7124 KB)

Supplementary File 7 Supplementary Figure S3 (TIF, 7153 KB)

Supplementary File 8 Supplementary Table S1 (XLS, 36 KB)
