# Supplementary figures and images for: A Global Expression Switch Marks Pachytene Initiation during Mouse Male Meiosis
Source: Genes (Basel). 2010 Dec 13;1(3):469–83. doi: 10.3390/genes1030469 (PMC3966219; doi:10.3390/genes1030469)

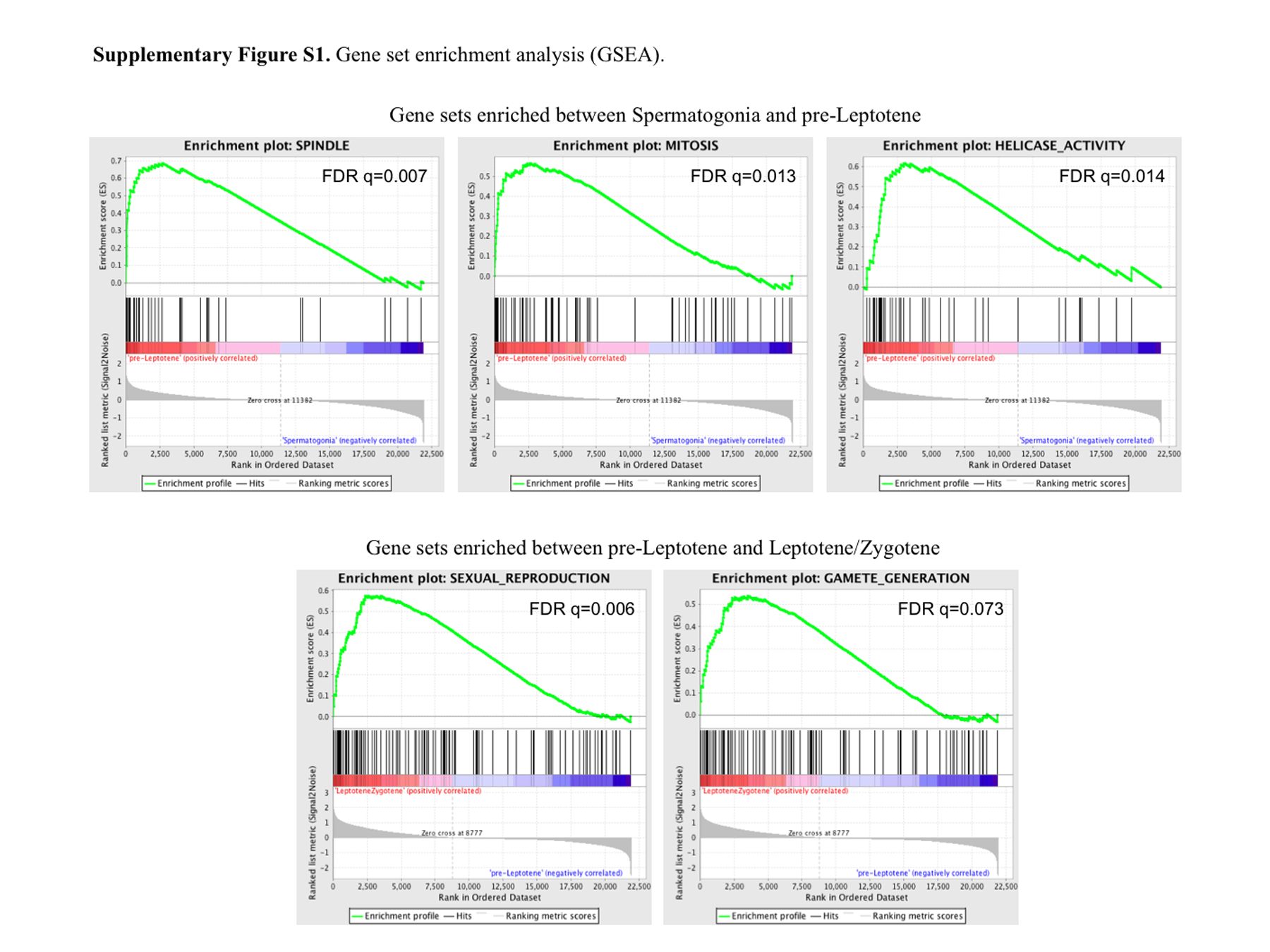

Supplement: Supplementary File 5 — Supplementary Figure S1 (TIF, 7141 KB) [file genes-01-00469-s005.tif]

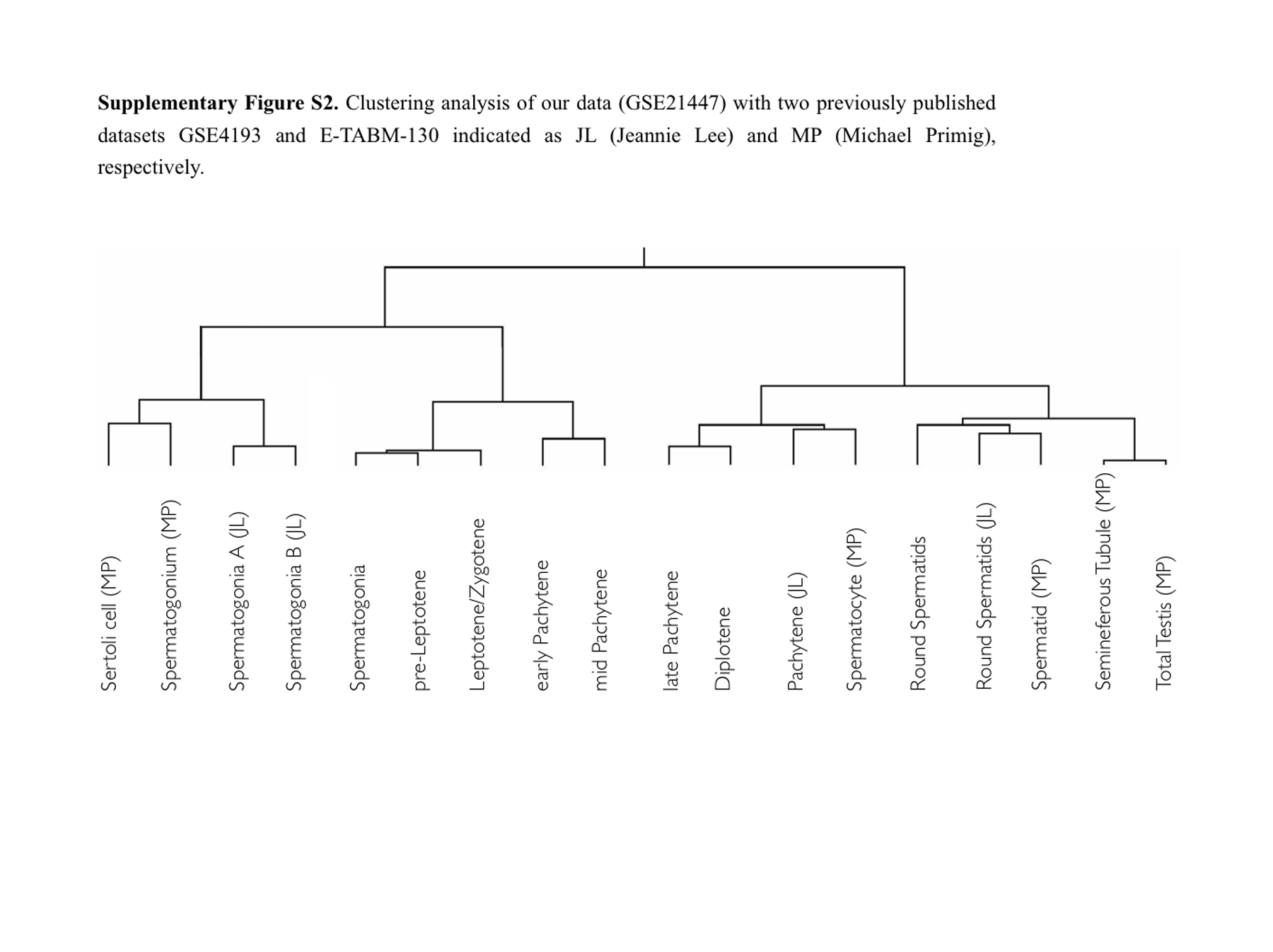

Supplement: Supplementary File 6 — Supplementary Figure S2 (TIF, 7124 KB) [file genes-01-00469-s006.tif]

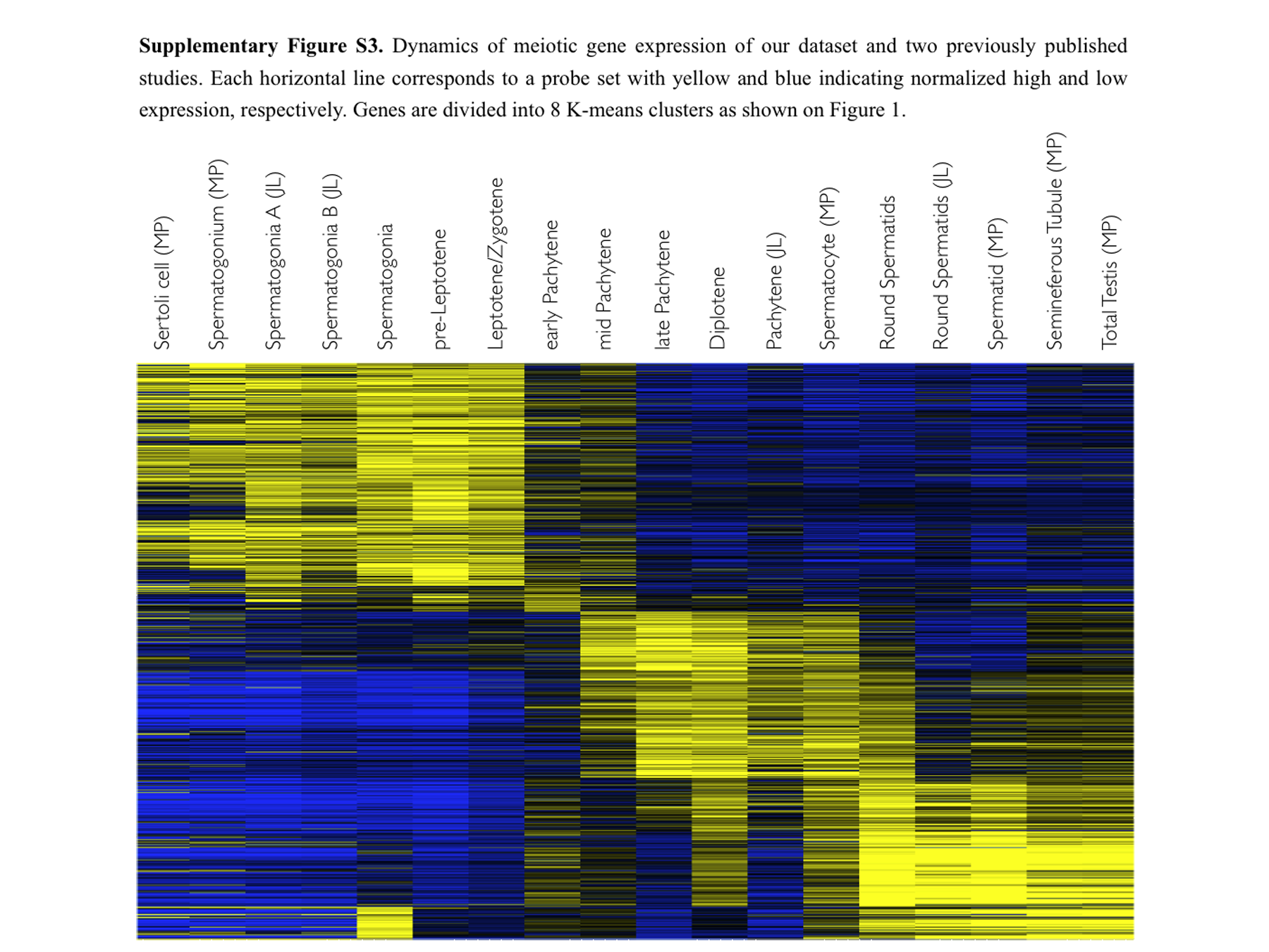

Supplement: Supplementary File 7 — Supplementary Figure S3 (TIF, 7153 KB) [file genes-01-00469-s007.tif]
